# Vacuolar proton-translocating ATPase is required for antifungal resistance and virulence of *Candida glabrata*

**DOI:** 10.1371/journal.pone.0210883

**Published:** 2019-01-23

**Authors:** Asuka Minematsu, Taiga Miyazaki, Shintaro Shimamura, Hiroshi Nishikawa, Hironobu Nakayama, Takahiro Takazono, Tomomi Saijo, Kazuko Yamamoto, Yoshifumi Imamura, Katsunori Yanagihara, Shigeru Kohno, Hiroshi Mukae, Koichi Izumikawa

**Affiliations:** 1 Department of Infectious Diseases, Nagasaki University Graduate School of Biomedical Sciences, Nagasaki, Japan; 2 Department of Respiratory Medicine, Nagasaki University Hospital, Nagasaki, Japan; 3 Faculty of Pharmaceutical Sciences, Suzuka University of Medical Sciences, Mie, Japan; 4 Department of Laboratory Medicine, Nagasaki University Graduate School of Biomedical Sciences, Nagasaki, Japan; Institute of Microbiology, SWITZERLAND

## Abstract

Vacuolar proton-translocating ATPase (V-ATPase) is located in fungal vacuolar membranes. It is involved in multiple cellular processes, including the maintenance of intracellular ion homeostasis by maintaining acidic pH within the cell. The importance of V-ATPase in virulence has been demonstrated in several pathogenic fungi, including *Candida albicans*. However, it remains to be determined in the clinically important fungal pathogen *Candida glabrata*. Increasing multidrug resistance of *C*. *glabrata* is becoming a critical issue in the clinical setting. In the current study, we demonstrated that the plecomacrolide V-ATPase inhibitor bafilomycin B_1_ exerts a synergistic effect with azole antifungal agents, including fluconazole and voriconazole, against a *C*. *glabrata* wild-type strain. Furthermore, the deletion of the *VPH2* gene encoding an assembly factor of V-ATPase was sufficient to interfere with V-ATPase function in *C*. *glabrata*, resulting in impaired pH homeostasis in the vacuole and increased sensitivity to a variety of environmental stresses, such as alkaline conditions (pH 7.4), ion stress (Na^+^, Ca^2+^, Mn^2+^, and Zn^2+^ stress), exposure to the calcineurin inhibitor FK506 and antifungal agents (azoles and amphotericin B), and iron limitation. In addition, virulence of *C*. *glabrata* Δ*vph2* mutant in a mouse model of disseminated candidiasis was reduced in comparison with that of the wild-type and *VPH2*-reconstituted strains. These findings support the notion that V-ATPase is a potential attractive target for the development of effective antifungal strategies.

## Introduction

Invasive candidiasis is one of the most frequent fungal infections among a wide spectrum of immunocompromised patients, with the in-hospital mortality rates reported to be as high as 20–40% even among patients who receive antifungal therapy [1]. The therapeutic options currently available to treat invasive candidiasis are limited to only four classes of antifungal agents: azoles, echinocandins, polyenes, and fluoropyrimidines. Further, the incidence rates of candidemia caused by non-*albicans Candida* species are increasing and antifungal resistance of these species has emerged as a serious problem in clinical practice [1–3]. The rise of multidrug resistance with unfavorable therapeutic outcome among *Candida glabrata* infections became a critical healthcare issue in the last decade [4–6]. Therefore, the development of novel antifungal strategies is urgently needed.

Recent studies highlight vacuolar proton-translocating ATPase (V-ATPase) as an attractive target for drug discovery (reviewed in [7]). V-ATPase is an ATP-driven proton pump present in the endomembranes of all eukaryotic organisms [8, 9]. In particular, this proton pump is present in fungal vacuolar membranes, where it plays an important role in the maintenance of intracellular ion homeostasis by maintaining acidic pH within cell [10–12]. The V-ATPase is composed of 14 subunits that form two domains, a membrane-integral V_0_ domain and a cytoplasmic V_1_ domain; and assembly factors, including Vph2 (Vma12), Vma21, Vma22, and Pkr1, are required for the assembly of a functional yeast V-ATPase [9, 13–15]. In *Saccharomyces cerevisiae*, V-ATPase synthesis and assembly are lost upon deletion of *VPH2*, leading to changes in ion sensitivity, including calcium sensitivity [16]. Previously, we demonstrated that V-ATPase also plays an important role in endogenous and exogenous oxidative stress response by regulating the expression and activity levels of the superoxide dismutase Sod2 and catalase Cta1, respectively, in *C*. *glabrata* [17].

Previous studies with mutant strains of *Histoplasma capsulatum*, *Cryptococcus neoformans*, and *C*. *albicans* lacking specific subunits of V-ATPase demonstrated that loss of V-ATPase function leads to vacuolar alkalinization and attenuation of *in vivo* virulence [18–21]. However, the link between V-ATPase function and virulence in *C*. *glabrata* has not been reported. In the current study, we investigated the effects of V-ATPase defect in *C*. *glabrata* on responses to various environmental stresses, antifungal resistance, and virulence.

## Materials and methods

### Strains, culture conditions, and compounds

*C*. *glabrata* strain CBS138 [22] was used as a wild-type control. *C*. *glabrata* Δ*vph2* deletion mutant lacking the entire *VPH2* open reading frame (NCBI accession no.: XP_448720, *Candida* genome database ID: CAGL0K11594g) and a *VPH2*-reconstituted strain, in which an intact *VPH2* was reintroduced at the native locus in the genome of the Δ*vph2* mutant, were constructed previously [17]. *C*. *glabrata* cells were propagated in yeast peptone dextrose (YPD) medium [1% (wt/vol) yeast extract, 2% (wt/vol) peptone, and 2% (wt/vol) glucose] or synthetic complete medium (SC) [0.67% (wt/vol) yeast nitrogen base with amino acids and 2% (wt/vol) glucose] at 30°C, unless otherwise specified. Media were solidified by the addition of 1.5% (wt/vol) agar. Fluconazole, voriconazole, amphotericin B, and FK506 were purchased from Sigma-Aldrich (St. Louis, MO). Bafilomycin B_1_ was purchased from Santa Cruz Biotechnology (Dallas, TX). Desferrioxamine (DFO) was purchased from EMD Chemicals (San Diego, CA) and bathophenanthroline disulfonate (BPS) was from MP Biomedicals (Solon, OH). Voriconazole, bafilomycin B_1_, and FK506 were dissolved in dimethyl sulfoxide and other compounds were dissolved in distilled water. Cell growth was not affected by exposure to the quantities of dimethyl sulfoxide used in the current study.

### Drug susceptibility assays

Susceptibility to fluconazole, voriconazole, and the V-ATPase inhibitor bafilomycin B_1_, alone or in combination, was examined by using broth microdilution test, essentially according to the Clinical and Laboratory Standards Institute (CLSI) M27-S4 protocol [23] and the previous report [24] with minor modifications. Briefly, *C*. *glabrata* cells were incubated in SC at 35°C for 48 h. The minimum drug concentration that inhibited cell growth by more than 80% relative to drug-free control was defined as the minimum inhibitory concentration (MIC). Fractional inhibitory concentration (FIC) was calculated by using the following formula: FIC for drug A = (MIC of drug A in combination with drug B)/(MIC of drug A alone). The sum of FIC for drug A and FIC for drug B was defined as the FIC index (FICI). Drug interaction was classified as synergistic if FICI was ≤0.5 [25].

Spot dilution test was performed as described previously [26]. Briefly, the density of logarithmic-phase cultures in SC was adjusted to the concentration of 2 × 10^7^ cells/ml. Serial 10-fold dilutions in SC were then prepared, and 5 μl of each dilution was spotted onto SC plates containing the test compound at the desired concentrations. Plates were incubated at 30°C for 48 h and photographed.

All sensitivity tests were performed on at least three separate occasions to ensure reproducibility.

### Staining of fungal cells

Vacuolar staining with the styryl dye *N*-(3-triethylammoniumpropyl)-4-(6-(4-(diethylamino)phenyl)hexatrienyl)pyridinium dibromide (FM4-64; Thermo Fisher Scientific, Molecular Probes, Eugene, OR) and the pH-sensitive fluorophore 2*N*,7*N*-bis-(2-carboxyethyl)-5-(and-6)-carboxyfluorescein acetoxymethyl ester (BCECF-AM; Thermo Fisher Scientific, Molecular Probes) was performed as described previously [27, 28] with few modifications. Briefly, logarithmic-phase cells of *C*. *glabrata* were washed and resuspended in SC broth (pH 5.0). FM4-64 was added to cell suspensions (final concentration: 5 μM) and the mixtures were incubated at 30°C for 15 min to stain vacuole membranes. Cells were washed in SC with agitation for 90 min and resuspended in SC. BCECF-AM was added to cell suspensions (final concentration: 18 μM) and incubated at 30°C for 60 min. Cells were washed twice in SC, and microscopic examination was performed immediately after washing. Images were acquired using a Carl Zeiss LSM780 confocal laser-scanning microscope and processed using ZEN 2011 software (Carl Zeiss, Jena, Germany). The excitation and emission parameters were as follows: 560 and 605 nm, respectively, for FM4-64; and 470 and 535 nm, respectively, for BCECF-AM.

### Virulence assay

Specific pathogen-free 8-week-old female BALB/c mice, weighing approximately 20 g, were purchased from Charles River Laboratories Japan (Yokohama, Japan). All mice had free access to food and water and were housed in a light–and temperature–controlled room at the Biomedical Research Center, Life Science Support Center, Nagasaki University. The health status of all mice was monitored at least daily throughout the experiments. All animal experiments were performed in full compliance with the Guide for the Care and Use of Laboratory Animals [29] and all institutional regulations and guidelines for animal experimentation, after pertinent review and approval by the Institutional Animal Care and Use Committee of Nagasaki University (protocol number 1407281164).

Logarithmic-phase cells of *C*. *glabrata* wild-type, Δ*vph2*, and *VPH2*-reconstituted strains were harvested, washed, and resuspended in sterile saline, and cell density was adjusted to 4 × 10^8^ cells/ml. The actual colony forming units (CFUs) used were confirmed by plating serial dilutions of the cell suspensions on YPD plates and incubating at 30°C overnight. Mice (*n* = 7 for wild-type, *n* = 9 for Δ*vph2*, and *n* = 8 for Δ*vph2* + *VPH2*, per experiment) were inoculated with 0.2 ml of each cell suspension via the lateral tail vein. Mice were euthanized by carbon dioxide-induced asphyxia 7 d after the injection, and the spleen, liver, and both kidneys were excised. The organs were homogenized in sterile saline using a Shake Master NEO (Bio Medical Science, Tokyo, Japan). The homogenates were appropriately diluted in sterile saline and plated on YPD agar. Colonies were counted after 48 h of incubation at 30°C and CFUs per organ were calculated. A *P*-value of <0.05 (Kruskal-Wallis test with Dunn’s post-test) was considered to represent statistical significance.

## Results

### Synergistic effects of azoles and the V-ATPase inhibitor bafilomycin B_1_ against *C*. *glabrata*

**Fig 1:**
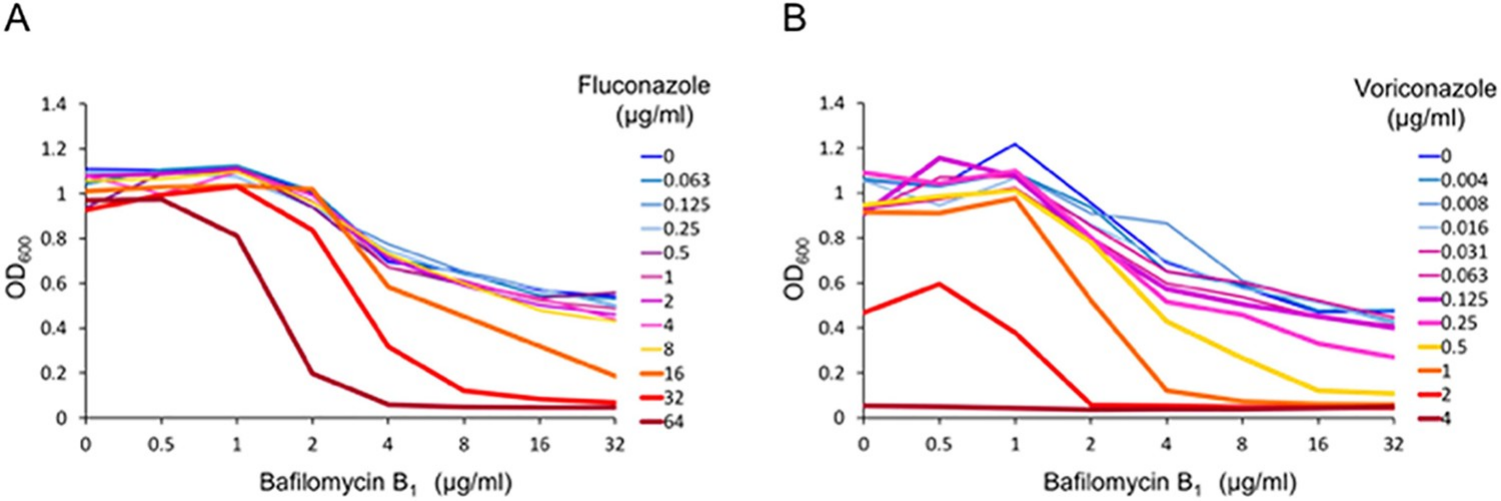
Synergistic effects of azole antifungals and the V-ATPase inhibitor bafilomycin B_1_ against *C*. *glabrata* wild-type strain. Checkerboard assay was performed using serial 2-fold dilutions of drugs. (A) Data for the combination of fluconazole and bafilomycin B_1_. (B) Data for the combination of voriconazole and bafilomycin B_1_. Plates were incubated at 35°C for 48 h and the optical density at 600 nm (OD_600_) was determined. The graphs are representative of three independent replicate experiments.

Azole antifungals, including fluconazole and voriconazole, inhibit the biosynthesis of ergosterol, the major component of fungal cell membrane, by targeting lanosterol 14α-demethylase encoded by *ERG11* [30]. V-ATPase is pharmacologically inhibited by the plecomacrolide bafilomycin B_1_, which binds to the V0 subunit of V-ATPase, and simultaneously interferes with ATP hydrolysis and proton transport [31, 32]. To examine the effect of bafilomycin B_1_ on azole susceptibility of *C*. *glabrata* wild-type strain, we performed a checkerboard assay using serial 2-fold dilutions of the drugs. In the assay, MICs of fluconazole, voriconazole, and bafilomycin B_1_ were determined to be >64, 4, and >32 μg/ml, respectively (Fig 1). FIC indices of the combination of fluconazole and bafilomycin B_1_, and the combination of voriconazole and bafilomycin B_1_ were 0.375 and 0.313, respectively, indicating synergistic effects of these azoles and bafilomycin B_1_ against *C*. *glabrata*.

### Deletion of the V-ATPase assembly factor gene *VPH2* leads to impaired vacuole acidification in *C*. *glabrata*

**Fig 2:**
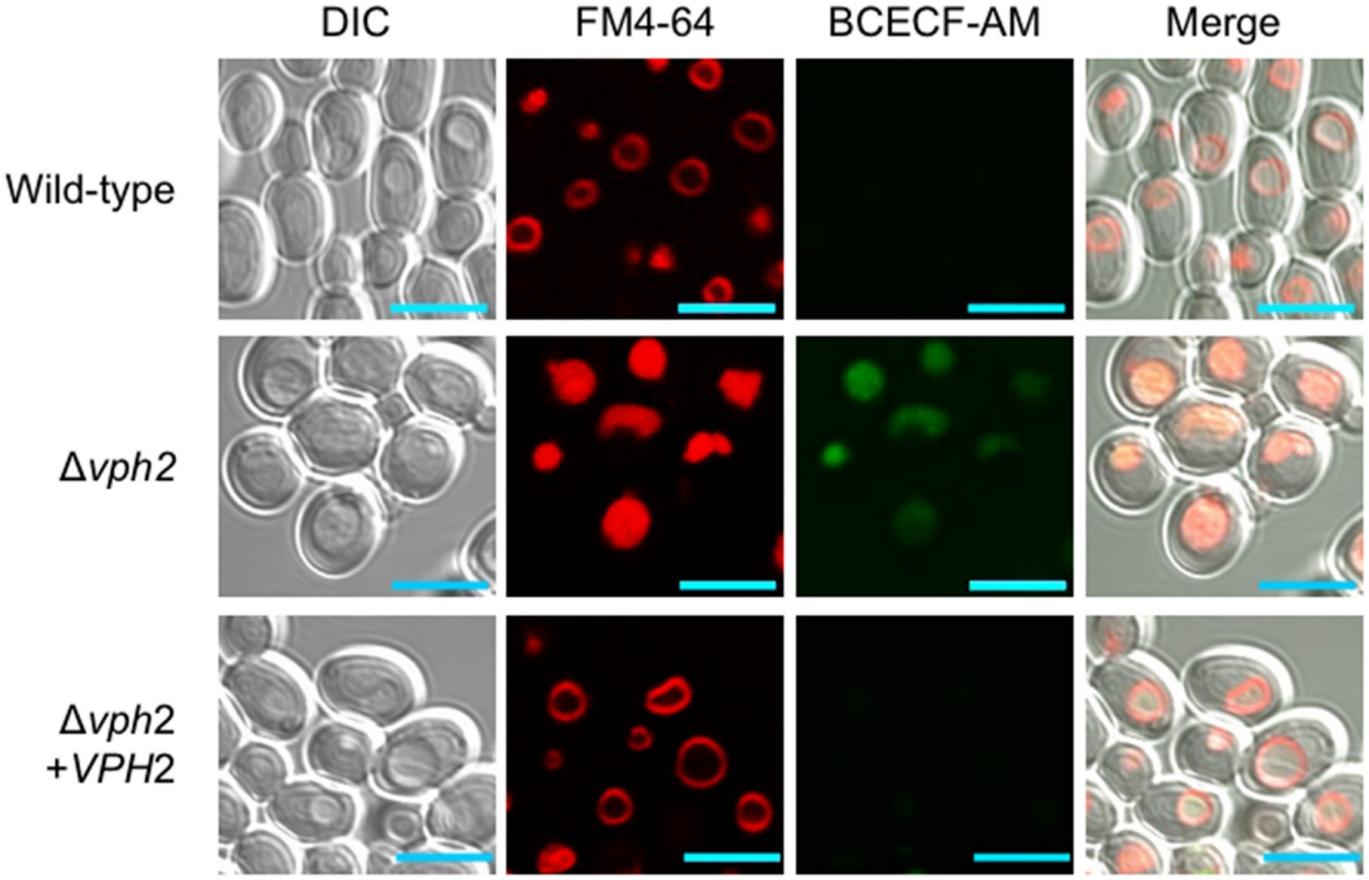
Vacuole staining. Logarithmic-phase cells of *C*. *glabrata* were prepared in SC broth. Vacuolar membranes were first stained with FM4-64. After washing, the pH-sensitive fluorophore BCECF-AM was added to cell suspensions. Note the accumulation of FM4-64 and BCECF-AM within the vacuole lumen of the Δ*vph2* mutant. Scale bars, 5 μm. The images are representative of three independent replicate experiments.

To investigate the role of V-ATPase in *C*. *glabrata* in detail, we analyzed the phenotype of the Δ*vph2* mutant, by comparing it with that of the wild-type and *VPH2*-reconstituted strains. First, *C*. *glabrata* cells were incubated with FM4-64, which selectively stains yeast vacuolar membranes and may be detected by red fluorescence [33, 34]. The wild-type and *VPH2*-reconstituted strains exhibited the typical ring-staining pattern of the vacuole membrane, while FM4-64 accumulated within the vacuole lumen in the Δ*vph2* mutant (Fig 2). The impaired trafficking of FM4-64 to the vacuolar membrane in the Δ*vph2* mutant was consistent with endocytosis defects demonstrated by *C*. *albicans vma* mutants [21] and *C*. *albicans* cells treated with fluconazole [28].

The cells were then labeled with the pH-sensitive fluorescent dye BCECF-AM. The dye labeled the vacuoles of the Δ*vph2* mutant but not those of the wild-type and *VPH2*-reconstituted strains (Fig 2). This indicated impaired vacuolar acidification in the Δ*vph2* mutant, as would be expected after loss of proton pump capacity.

### Loss of Vph2 results in increased fungal sensitivity to various environmental stresses

**Fig 3:**
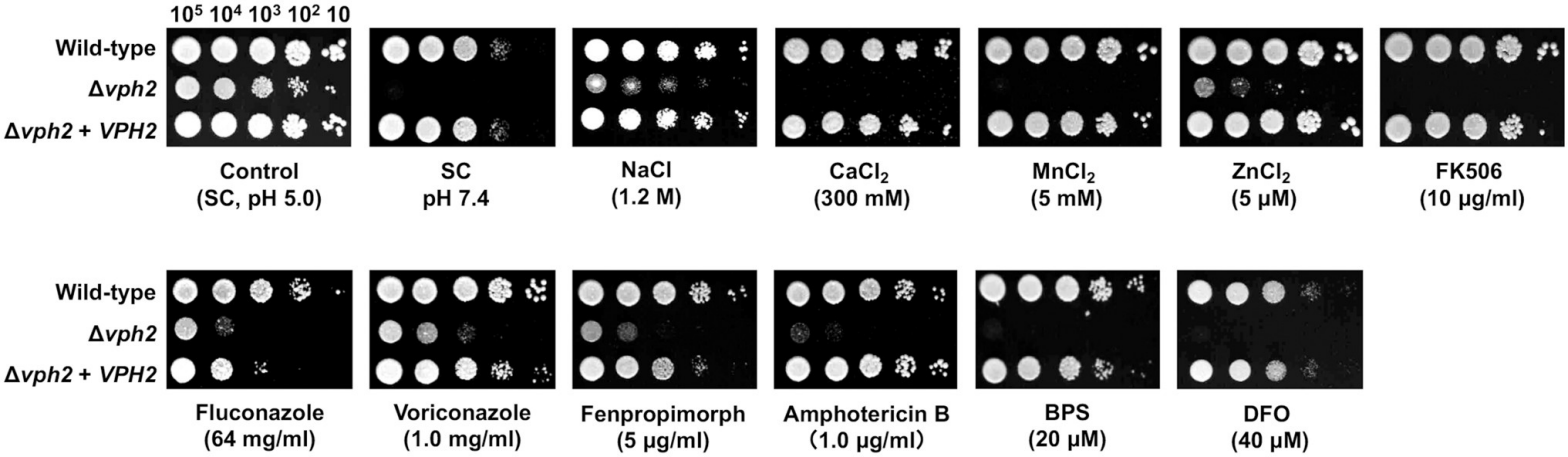
Spot dilution assay. Serial 10-fold dilutions of logarithmic-phase cells of *C*. *glabrata* were spotted onto SC plates containing the indicated compounds at the specified concentrations. Plates were incubated at 30°C for 48 h and photographed. The images are representative of three independent replicate experiments. BPS, bathophenanthroline disulfonate; and DFO, desferrioxamine.

The phenotype of the Δ*vph2* mutant was further examined by spot dilution assays. In agreement with the notion of impaired vacuole acidification even under acidic conditions (Fig 2), the Δ*vph2* mutant exhibited a growth defect at pH 5.0 and was unable to grow at pH 7.4 (Fig 3). The Δ*vph2* mutant also displayed increased sensitivity to ion stress induced by excess of NaCl, CaCl_2_, MnCl_2_, and ZnCl_2_ in the growth medium.

The Ca^2+^/calmodulin-dependent protein phosphatase calcineurin plays a critical role in maintaining intracellular ion homeostasis and cell integrity. Further, simultaneous loss of certain subunits of V-ATPase and calcineurin is synthetically lethal in *S*. *cerevisiae* [35, 36]. In agreement with the findings for *S*. *cerevisiae*, the *C*. *glabrata* Δ*vph2* mutant was unable to grow in the presence of the calcineurin inhibitor FK506 (Fig 3). In addition to fluconazole and voriconazole, the Δ*vph2* mutant displayed increased susceptibility to fenpropimorph, which inhibits C-8 sterol isomerase (Erg2) and C-14 sterol reductase (Erg24) in the ergosterol biosynthesis pathway [37, 38], and amphotericin B, which directly targets ergosterol [39]. Finally, the Δ*vph2* mutant also exhibited growth defects under iron-limited conditions induced by the inclusion of the bacterial siderophore DFO or the Fe^2+^-chelator BPS in the growth medium.

### Loss of Vph2 results in reduced fungal virulence in a murine model of disseminated candidiasis

**Fig 4:**
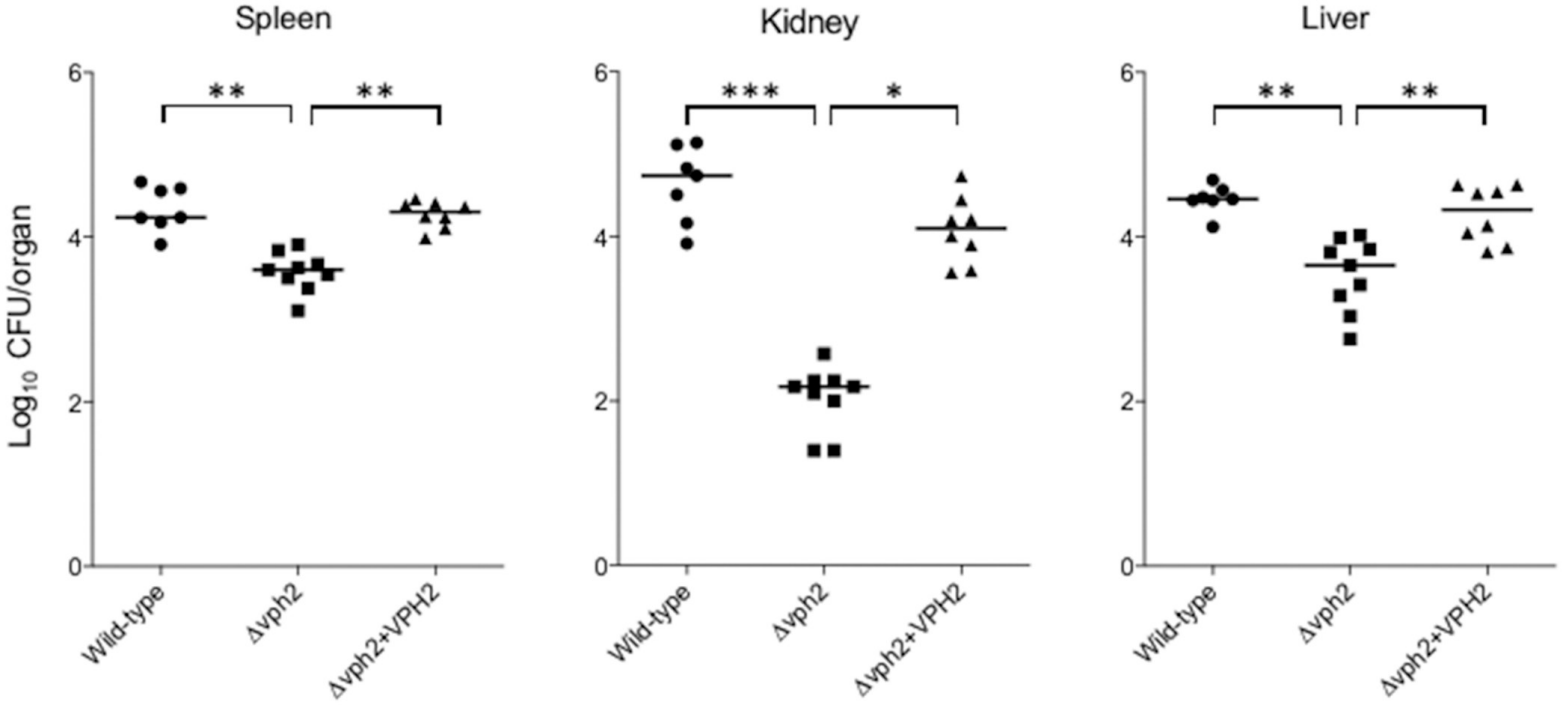
Fungal virulence in a mouse model of disseminated candidiasis. Eight-week-old female BALB/c mice were intravenously inoculated with 8 × 10^7^ cells of each *C*. *glabrata* strain (wild-type, *n* = 7; Δ*vph2*, *n* = 9; and Δ*vph2* + *VPH2*, *n* = 8; per experiment). The mice were sacrificed 7 d after inoculation and CFUs per organ in specific organs were determined. The geometric mean is shown as a bar. Data representative of two independent experiments are shown. The *C*. *glabrata* strains used were: wild-type (CBS138), filled circles; Δ*vph2* mutant, squares; and *VPH*2-reconstituted strain, triangles. *P < 0.05, **P < 0.01, ***P < 0.001 (Kruskal-Wallis test with Dunn’s post-test).

The effect of *VHP2* deletion on the virulence of *C*. *glabrata* was examined using a mouse model of disseminated candidiasis. No mice died prior to euthanasia in the experiments. Fungal burdens in the examined organs of immunocompetent mice infected with the Δ*vph2* mutant were significantly lower than those in mice infected with the wild-type or *VPH2*-reconstituted strains (Fig 4). This suggested that V-ATPase plays an important role in the virulence in *C*. *glabrata*.

## Discussion

Overcoming the antifungal resistance of *C*. *glabrata* in the clinical setting is a pressing issue. In the current study, we demonstrated the synergistic effect of the V-ATPase inhibitor bafilomycin B_1_ and azole antifungals against a *C*. *glabrata* wild-type strain. Azole antifungals exert an antifungal effect partly by impairing vacuolar acidification, since ergosterol is required for V-ATPase to function efficiently [28]. The concurrent disruption of ergosterol and V-ATPase was induced by exposing the Δ*vph2* mutant to ergosterol inhibitors, leading to the severe growth impairment of the Δ*vph2* mutant (Fig 3). Some *C*. *glabrata* Δ*vph2* phenotypes were anticipated based on the published findings in *S*. *cerevisiae* and *C*. *albicans*. However, in the current study, we demonstrated for the first time that the loss of Vph2 in *C*. *glabrata* results in a V-ATPase defect, which leads to impaired vacuolar pH homeostasis and increased sensitivity to a variety of environmental stresses, as well as attenuated virulence in mice. The growth defect of the Δ*vph2* mutant could contribute to the enhanced susceptibility to diverse drugs tested and decreased virulence.

The Δ*vph2* mutant was unable to grow under iron-limiting conditions. Requirement of V-ATPase for iron homeostasis was also demonstrated in *H*. *capsulatum* [19]. Iron acquisition and iron homeostasis are important virulence factors in pathogenic fungi. For example, *C*. *albicans* must obtain hemoglobin iron to survive under the iron-limiting conditions in host tissues, and functional V-ATPase is required for iron acquisition in the microorganism [40].

Targeting a conserved protein that plays an essential role in human and fungal cells is challenging as it entails averting host toxicity. For instance, V-ATPase is present in the renal tubules and osteoclasts in mammals, including human [8]. However, although V-ATPase is highly conserved in eukaryotes, some major differences between mammalian and fungal V-ATPases exist, particularly with respect to the isoform composition of subunits and in the regulation of complex disassembly [41–45]. The different numbers and types of isoforms have been developed for most subunits of the mammalian V-ATPase [7]. The sequence conservation between *S*. *cerevisiae* and human V-ATPase subunits is 51–60% similarity and 31–41% identity, depending on the subunit and isoform [44]. *C*. *glabrata VPH2* encodes a putative protein of 209 amino acids, with a molecular mass of 23.4 kDa. The deduced amino acid sequences of *C*. *glabrata VPH2* share 61.8% similarity and 41.9% identity with those of *S*. *cerevisiae VPH2* (NCBI Gene ID 853741, NCBI accession no. CAA81960), but only 32.6% similarity and 18.1% identity with those of a human homolog (TMEM199: NCBI Gene ID 147007, NCBI accession no. NP_689677) (S1 Fig). These different features could potentially be exploited to selectively target V-ATPase of pathogenic fungi.

In conclusion, in the current study, we provided evidence that disruption of *C*. *glabrata* V-ATPase function by deleting *VPH2* impairs the fungal response to various environmental stresses and results in the attenuation of virulence of this clinically important fungal pathogen, supporting the notion that V-ATPase is an attractive antifungal target.

## Supporting information

S1 Fig Sequence alignment of the deduced amino acids of *C*. *glabrata VPH2* with those of *S*. *cerevisiae VPH2* and a human homolog TMEM199. Identical and similar amino acids are shown as darkly shaded and lightly shaded regions, respectively. GenBank accession number: *C*. *glabrata VPH2*, XP_448720; *S*. *cerevisiae VPH2*, CAA81960; and TMEM199, NP_689677.
(TIF)
