# Supplementary figures and images for: Vacuolar proton-translocating ATPase is required for antifungal resistance and virulence of Candida glabrata
Source: PLoS One. 2019 Jan 23;14(1):e0210883. doi: 10.1371/journal.pone.0210883 (PMC6343876; doi:10.1371/journal.pone.0210883)

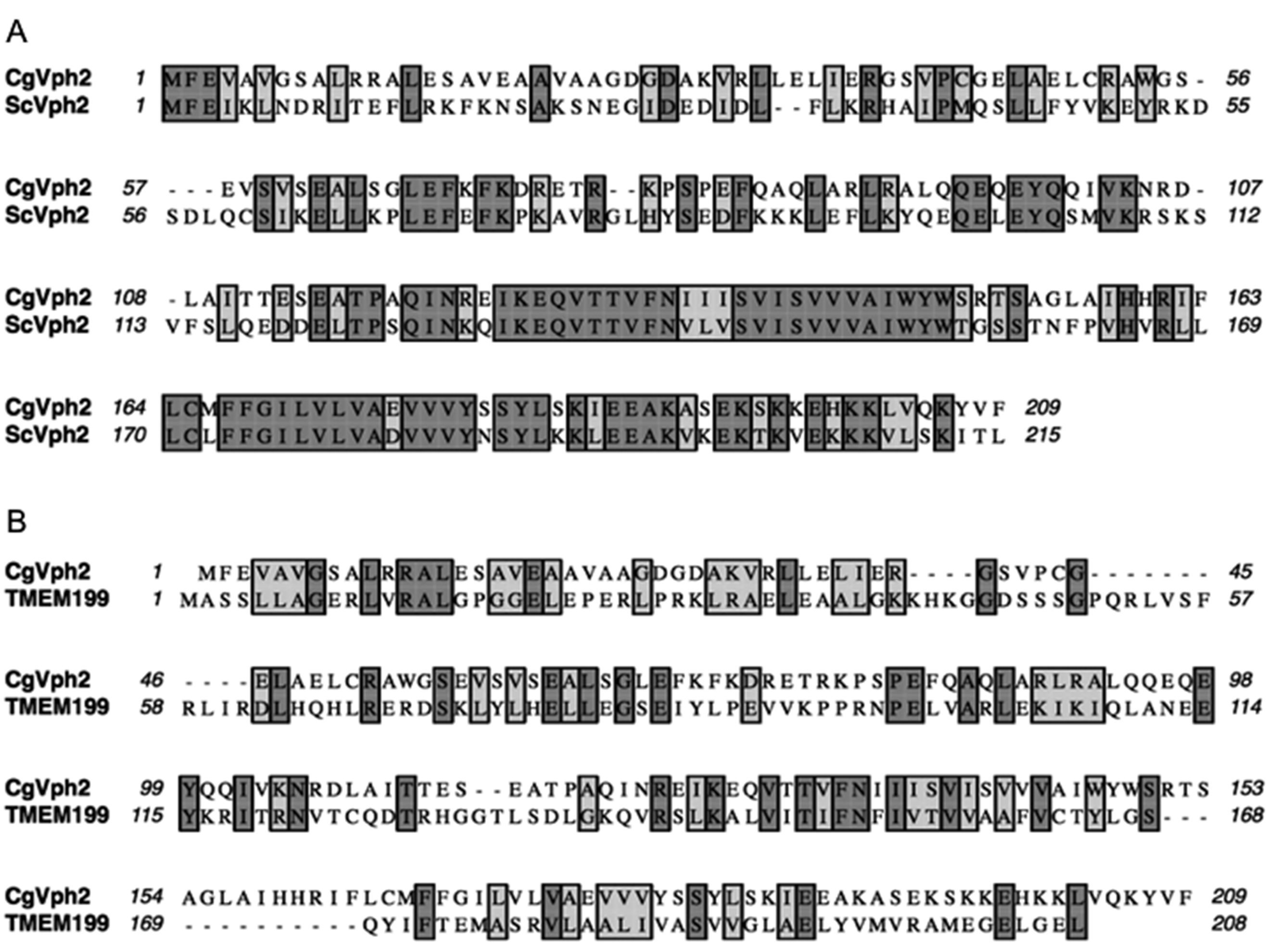

Supplement: S1 Fig — Identical and similar amino acids are shown as darkly shaded and lightly shaded regions, respectively. GenBank accession number: C. glabrata VPH2, XP_448720; S. cerevisiae VPH2, CAA81960; and TMEM199, NP_689677. (TIF) [file pone.0210883.s001.tif]
